# Sport-2-Stay-Fit study: Health effects of after-school sport participation in children and adolescents with a chronic disease or physical disability

**DOI:** 10.1186/s13102-015-0016-7

**Published:** 2015-10-06

**Authors:** Maremka Zwinkels, Olaf Verschuren, Kristel Lankhorst, Karin van der Ende-Kastelijn, Janke de Groot, Frank Backx, Anne Visser-Meily, Tim Takken, on behalf of the Sport-2-Stay-Fit study group

**Affiliations:** 1 Center of Excellence for Rehabilitation Medicine and Brain Center Rudolf Magnus, De Hoogstraat Rehabilitation and University Medical Center Utrecht, Utrecht, The Netherlands; 2 Partner of Shared Utrecht Pediatric Exercise Research (SUPER) Lab, Utrecht, The Netherlands; 3 Research Group Lifestyle and Health, Institute of Human Movement Studies, University of Applied Sciences, Utrecht, The Netherlands; 4 Department of Rehabilitation, Nursing Science and Sports, University Medical Center Utrecht, Utrecht, The Netherlands; 5 Child Development and Exercise Center, University Medical Center Utrecht, Wilhelmina Children’s Hospital, P.O. Box 85090, 3508 AB Utrecht, The Netherlands

**Keywords:** Children, Adolescents, Physical fitness, Health, Chronic disease, Physical disability, Interval training, Exercise, Sport participation

## Abstract

**Background:**

Children and adolescents with a chronic disease or physical disability have lower fitness levels compared to their non-disabled peers. Low physical fitness is associated with reduced physical activity, increased cardiovascular diseases, and lower levels of both cognitive and psychosocial functioning. Moreover, children and adolescents with a chronic disease or physical disability participate less in both recreational and competitive sports. A variety of intervention studies have shown positive, but only temporary, effects of training programs. Next to issues related to the chronic condition itself, various personal and environmental factors play a key role in determining the extent to which they participate in sports or physical activities. Due to these barriers, sport participation in the immediate after-school hours seems to be a feasible solution to get these children and adolescents physical active structurally. To investigate if an after school sport program can sustain the positive effects of an intervention, a standardized interval training will be given to improve physical fitness levels. High-intensity Interval Training (HIT) is superior to moderate-intensity continuous training in improving physical fitness in patients with chronic diseases. Therefore, the Sport-2-Stay-Fit study will investigate whether after school sport participation can increase the sustainability of a HIT program in children and adolescents with a chronic disease or physical disability.

**Methods:**

The Sport-2-Stay-Fit study is a clinical controlled trial. A total of 74 children and adolescents in the age of 6–19 years with a chronic disease or physical disability will be included. This could be either a cardiovascular, pulmonary, metabolic, musculoskeletal or neuromuscular disorder. Both children and adolescents who are ambulatory or propelling a manual wheelchair will be included. All participants will follow a HIT program of eight weeks to improve their physical fitness level. Thereafter, the intervention group will participate in sport after school for six months, while the control group receives assessment only. Measurements will take place before the HIT, directly after, as well as, six months later. The primary objective is anaerobic fitness. Secondary objectives are agility, aerobic fitness, strength, physical activity, cardiovascular health, cognitive functioning, and psychosocial functioning.

**Discussion:**

If effective, after school sport participation following a standardized interval training could be implemented on schools for special education to get children and adolescents with a chronic disease or physical disability active on a structural basis.

**Trial registration:**

This trial is registered at the Dutch Trial Register #NTR4698.

## Background

Children and adolescents with a chronic disease or physical disability have lower fitness levels compared to their non-disabled peers [1–6]. Low physical fitness is highly associated with reduced physical activity, increased cardiovascular diseases and overall mortality in healthy populations [7–10]. Additionally, cardiovascular risk factors such as dyslipidemia, hypertension, hyperinsulinemia or insulin resistance, and obesity often exist in children and young adults [11, 12]. To be healthy later in life, it is assumed that children and adolescents should be physically active on a structural basis.

At the same time, children and adolescents who are healthy [13] or physical active [14, 15] perform better at school. Moreover, childhood physical fitness is associated with higher levels of cognition [15–17]. Physical fitness level is a strong predictor in children and adolescents of school performance and level of cognition one year later [18, 19]. School performance has improved in children and adolescents who are typically developing by intervention programs stimulating physical activity and sports [20, 21]. However, this relationship between physical fitness and cognition level is still unknown in children and adolescents with a chronic disease or physical disability.

Another positive effect related to participation in sports is improving quality of life. This relation has been found in children and adolescents with cerebral palsy [22, 23]. In addition, children with lower motor skills have a lower global self-perception than their well-coordinated peers [24]. Perceptions of physical competence are strong predictors of self-perception among children with motor coordination difficulties [24, 25]. Successful performance experiences are the strongest means of changing self-perception; sport participation [26, 27]. Short-term sport interventions, however, did not significantly improve the self-perception of children with a physical disability although motor skills did improve [28, 29]. The long-term effect of sport participation on self-perception in children and adolescents with a chronic disease or physical disability still has to be established.

It can be assumed that the benefits of participating in sports are universal for all children, including those with a chronic disease or physical disability. However, they participate less in competitive and recreational sports compared to their non-disabled peers [30, 31]. A variety of interventions have shown that training programs improved physical fitness levels and participation in physical activities or sports. However, the positive effects following the training program did never sustain [23, 32, 33]. Therefore, more recently a shift in focus has moved from interventions to improve fitness to interventions to increase physical activity [30, 34].

For children and adolescents with a chronic disease or physical disability it is difficult to participate in sports or physical activities. Next to issues related to the chronic condition itself, various personal and environmental factors play a key role [35–37]. The most important barriers are: physical ability of the child, decreased motivation, lack of awareness of sports possibilities and lack of access to transportation. Most of these barriers can possibly be eliminated when an adapted sports program is provided at school. Interventions to promote sports in healthy young people conducted in the hours after school are effective in improving physical fitness, physical activities and health [38, 39]. A few special schools in the Netherlands are experienced in organizing and facilitating an after-school sports program. Although they anecdotally notice a lot of progress in these children, they have never measured the effect accurately in a scientific and systematic way.

To investigate if an after school sport program can sustain physical fitness level, health, and both cognitive and psychosocial functioning, a baseline fitness level is needed. Standardized exercise training with prescribed work-rest ratios, intensity, volume and time are more effective in improving physical fitness, especially short term [23, 32, 33]. In this regard, high-intensity Interval Training (HIT) is superior to moderate-intensity continuous training in improving physical fitness in patients with chronic diseases [40–42]. Furthermore, HIT is feasible for these children and adolescents [23]. Therefore, prior to the after-school sport program, a HIT program will be given to improve physical fitness level.

The current study, denoted as the Sport-2-Stay-Fit study, will investigate whether an after school sports program can increase the sustainability of a HIT program in children and adolescents with a chronic disease or physical disability. In addition, sport participation in these children may have positive effects on cardiovascular health, and both cognitive and psychosocial functioning. While these positive relationships have been reported in children and adolescents who are typically developing, the benefits are not established in those with a chronic disease or physical disability.

## Methods

### Design

**Fig. 1:**
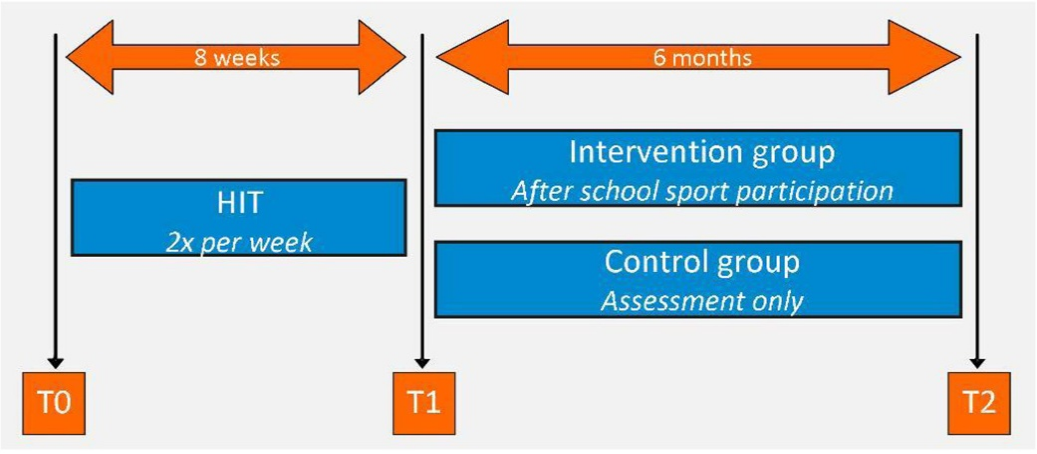
Schematic research design of the Sport-2-Stay-Fit study. HIT: High-intensity interval training

The Sport-2-Stay-Fit study is a controlled clinical trial. All children and adolescents will participate in an eight week HIT program to improve their physical fitness level. Thereafter, the intervention group will participate in an after-school sports program for six months, while the control group will receive assessments only. Outcome measures will be assessed at baseline (T0), immediately after eight weeks of HIT (T1), and at completion of six months intervention (T2) (Fig. 1). Similar outcome measures will be evaluated at T0, T1 and T2 except for physical activity at T1, because no effect or changed difference between groups was expected [43, 44]. The current study is part of a larger project in which children and adolescents with a chronic disease or physical disability who are active in sports will be compared to their non-sporting peers [45].

### Setting

In total 74 participants will be recruited from four schools for special education in the Netherlands: Ariane de Ranitz|De Hoogstraat Rehabilitation in Utrecht, Lichtenbeek in Arnhem, Mariendael in Arnhem, and Heliomare in Wijk aan Zee. Schools experienced in organizing and facilitating an after school sports program, will be included in the intervention group. To prevent bias, the control group will only be included at schools where no after school sport is provided. In this way, the research design is close to reality and participants are not unethically prohibited to sport at school. Both ethics approval and administrative site approvals were granted by the Medical Ethical Committee of UMC Utrecht in the Netherlands (#14–118). Additionally, all parents, and participants from 12 years of age, have to provide informed consent prior to study initiation.

### Participants

Children and adolescents between the age of 6 and 19 with a chronic disease or physical disability will be included. This could be either cardiovascular, pulmonary, musculoskeletal, metabolic or neuromuscular disorders. Both children and adolescents who are ambulatory or propelling a manual wheelchair are asked to participate. They may participate in sports at most once a week during leisure time in the preceding three months or have a treatment goal on fitness level prescribed by their physician. Participants have to understand simple commands and are able to perform the physical fitness tests. Children and adolescents using electric wheelchair or having progressive diseases will be excluded. In addition, during the length of the study, children and adolescents are not allowed to participate in other research projects which might influence current study results.

### Intervention

All participants will follow a training program of eight weeks to increase their physical fitness level. A HIT program will be given twice a week at school by a physical educator and/or a physical therapist. Every training session will consist of a prescribed intensity, volume and time (Table 1). According to the review of Baquet et al. the 30 seconds all out approach for interval training is recommended in youth [46]. An active recovery has been suggested in order to effectively aid the process of lactate removal [47]. Sitting will therefore be forbidden during the interval training and children will be encouraged to motivate and support the others while exercising. After eight weeks of HIT, the experimental group will participate in an after school sport program for six months. This is an extra sport lesson additional to the regular physical education schedule. During regular physical education half of the time is spend on skill practice, physical education knowledge and management in children with a physical disability [48]. In contrast, sport is about being physical active, playing the game, and being competitive.

**Table 1:** Schematic design of the HIT program. HIT: High-intensity interval training

	Week 1	Week 2	Week 3	Week 4	Week 5	Week 6	Week 7	Week 8
Frequency (p/w)	2	2	2	2	2	2	2	2
Duration	30 sec	30 sec	30 sec	30 sec	30 sec	30 sec	30 sec	30 sec
Intensity	max	max	max	max	max	max	max	max
Work:rest ratio	1:4	1:4	1:4	1:4	1:3	1:3	1:3	1:3
Repetitions	8	8	10	10	12	12	12	12
Total time	20 min	20 min	25 min	25 min	24 min	24 min	24 min	24 min

### Outcome measures

A general screening questionnaire will be used to inquire about possible factors influencing the outcomes: age, sex, physical activities, health status, injuries. The following outcome measures can be divided: physical fitness, cardiovascular health, physical activity, cognitive functioning, and psychosocial functioning (Table 2).

**Table 2:** Overview of the outcome measures of the Sport-2-Stay-Fit

Outcome measure	Parameter	Measurement
Physical Fitness	Anaerobic fitness	Muscle Power Sprint Test (MPST)
	Agility	10x5 meter sprint
	Aerobic fitness	Shuttle run/ride test (SRT)
	Strength	Grip strength
		Reverse curl
		Seated push up
		Standing broad jump/One stroke push
	Flexibility	Modified Apley test
		Modified Thomas test
Cardiovascular Health	Morphologic	Length, weight, BMI
		Bioelectrical impedance analysis
	Metabolic	Sphygmomanometer
		Arteriograph
		Finger puncture
Physical Activity	Modality	Activ8
		Activity diary
Cognitive functioning	School performance	Type of education
		Educational achievement test
	Attention	Bourdon-Vos
		Cancellation task
		Capture task
Psychosocial functioning	Self-perception	Self-Perception Profile for Children (SPPC)
	Quality of life	Disabkids
	Self-efficacy	Exercise Self-efficacy Scale (ESES)

### Primary outcomes

Anaerobic fitness is the primary outcome measure in the current study, because daily physical activity in children most often consists of brief, intermittent bouts of intense movement [49, 50]. Consequently, the anaerobic fitness of healthy individuals involved in recreational sport programs is significantly better than that of those individuals who are not involved in any activities [51]. The Muscle Power Sprint Test (MPST) will be used to evaluate anaerobic fitness. This test is suitable and validated for both children and adolescents who are typically developing, and with cerebral palsy who are ambulant or self-propel a manual wheelchair [6, 52–54]. Subjects have to complete six 15-meter runs at a maximum pace. The MPST is an intermittent sprint test, in which the child starts and stops at standardized intervals. Power output will be calculated for each of the six sprints. Peak power will be defined as the highest calculated power, while mean power will be defined as average power over the six sprints.

### Secondary outcomes

#### Agility

The 10x5 meter sprint test will be performed to asses agility, either while running or propelling a wheelchair. This is a reliable field test to measure agility in children with cerebral palsy [52, 53]. During this test, the child has to sprint as fast as possible for 10 times in between two lines that are five meters apart. Since there is no resting period, the participant has to turn as fast as possible.

#### Aerobic fitness

Aerobic fitness, generally expressed as peak oxygen uptake (VO_2_peak), is a good predictor of cardiovascular risk [55, 56]. VO_2_peak will be measured with a field test. Shuttle run/ride test (SRT) is a field test in which a participant runs, walks or rides between two markers. The SRT starts at 1.5 km/h, 2 km/h or 5 km/h and has shown to be a valid and reliable test in children with cerebral palsy measuring aerobic fitness [57, 58]. The children have to adjust their running, walking or wheelchair propulsion pace to the beep-signals, until they fail to reach the line two times in a row, despite encouragements. Subsequently, a participants mode of locomotion will be identified in order to apply a proper testing protocol (Table 3). Based on clinical observations, participants will be divided into four groups: subjects who are able to run, subjects who walk independently but are not able to run, subjects using walking devices, and wheelchair users. Running is defined in the ICF as moving with quick steps so that both feet may be simultaneously off the ground [59].

**Table 3:** Overview of different SRT protocols. SRT: shuttle run/ride test

Participants	Protocol	Distance	Starting pace	Increasement per min
Able to run	SRT I	10-meter line	5.0 km/h	0.25 km/h
Walk independently (not able to run)	SRT II	10-meter line	2.0 km/h	0.25 km/h
Using walking devices	SRT II	7.5-meter square	1.5 km/h	0.19 km/h
Wheelchair users	SRT II	10-meter line	2.0 km/h	0.25 km/h

Each test will last until volitional exhaustion and protocols will be chosen with the aim to achieve a total exercise time between 6 and 15 min [60, 61]. The shuttle tests will be evaluated with the Cortex Metamax 3X (Samcon bvba, Melle, Belgium), a valid and reliable cardio-pulmonary exercise testing system [62–64]. The Cortex Metamax 3X consists of a facemask, transmitting unit attached to a harness containing oxygen and carbon dioxide analysers, and a receiving unit. Metabolic stress test software (Metasoft Studio) will be used to measure minute ventilation, oxygen uptake (VO_2_), carbon dioxide production (VCO_2_), heart rate, and respiratory exchange ratio (RER = VCO_2_/VO_2_) every 10 seconds.

#### Strength

To assess explosive functional strength, the standing broad jump and one stroke push will be performed in children who are ambulatory and propel a manual wheelchair, respectively. Lower limbs strength will be measured by the distance jumped with two legs together from standing position [65] and upper limbs strength by measuring the distance covered in a wheelchair by one push [66]. The following tests are chosen from the Brockport Physical Fitness Test, which was designed to test fitness of youths with various disabilities and is both health related and criterion referenced. Grip strength represents the general strength in healthy children [67]. The grip strength of the dominant hand will be tested through the use of a hand held dynamometer, as described by Beenakker et al. [68] The reverse curl is designed as a measure of hand, wrist, and arm strength [69]. In this test, the participant attempts to pick up a 0.5-kg dumbbell with the preferred arm while seated in a chair or wheelchair. The seated push up is designed to measure upper-body strength and endurance [69]. The participant attempts to perform a seated push-up and holds it for up to 20 seconds.

#### Flexibility

To test flexibility, the Modified Apley Test and the Modified Thomas Test were chosen from the Brockport Physical Fitness Test. To measure upper-body flexibility with the Modified Apley test, participants attempt to reach back and touch the superior medial angle of the opposite scapula with one hand [69]. The Modified Thomas Test is designed to assess the length of participant’s hip flexor muscles: iliopsoas and rectus femoris muscles [69].

#### Cardiovascular health

BMI, body mass index, will be calculated as weight (kg)/length (m)^2^. Height will be measured in standing position in case of ambulant subjects and supine in case of wheelchair bound persons. When a person has spastic legs, arm span width will be measured and corrected as described by Dosa et al. 2009 [70]. Fat free mass will be determined with bioelectrical impedance analysis (BIA), using the Bodystat Quadscan 4000 (Euromedix, Leuven, Belgium). BIA is a non-invasive test comparing conductivity and resistance in the body to distinguish lean body mass and fat [71].

Increased arterial stiffness is associated with a higher likelihood of cardiovascular disease [72]. Arterial stiffness exists of two independent values. The Pulse Wave Velocity (PWV) is measured as the speed at which an aortic pulse travels; increased speed will indicate stiffer arteries. The Augmentation Index (AIX) provides information on the peripheral resistance of the endothelial vessels. Blood pressure and arterial stiffness measurements will be performed using arteriography. The arteriograph (Litra, Amsterdam, the Netherlands) will measure blood pressure, as well as arterial stiffness, using oscillometric tonometry. This method is valid compared with the golden standard: invasive measurement during cardiac catheterization [73]. Each subject will rest for at least 10 min prior to the recording. The measurement will be executed using an inflatable cuff at the right upper arm. The participants may not move or talk during the measurement.

To determine metabolic parameters, a finger puncture will be performed to measure cholesterol, low density lipoprotein, high density lipoprotein, glucose and triglyceride. The finger puncture will be performed using a Cholestech LDX analyzer (Mediphos Medical Supplies BV, Renkum, the Netherlands). Participants may not eat or drink for three hours prior to this procedure.

#### Physical activity

An Activ8 (2M Engineering, Valkenswaard, the Netherlands) accelerometer will be used to measure the type, duration, frequency and intensity of physical activity in daily life. The system is valid and reliable to detect the type, duration, frequency and intensity of physical activity in persons able to walk [74]. Both children who are ambulatory and wheelchair dependent wear the Activ8 on their upper leg for five consecutive days: three weekdays and two weekend days. Wearing the activity monitor will be no burden for the participants; all activities in daily life and during sport activities can be performed. The general activity pattern, incidence and type of injury during the last 3 months will be measured by a questionnaire.

#### Cognitive functioning

Childhood physical fitness is associated with higher levels of cognition; attention and school performance [15, 16, 75]. For quantifying school performance, a Dutch educational achievement test will be used, which should be administered at school annually. All scores can be converted to a didactic age equivalent. In addition, a more sensitive outcome measure has been chosen to detect changes longitudinally. Cognition was operationalized into three components: sustained attention, search efficiency and distractibility. All tasks will be performed on a tablet (Asus Eee Slate Tablet, with a 12.1 inch display and clock speed of 1.33 GHz) overcoming coordination problems and guaranteeing detection of subtle improvements in performance. Sustained attention will be measured using an adapted digitalized version of the Bourdon-Vos task [76, 77]. The Bourdon-Vos Test is a cancellation test requiring high-speed visual selectivity and a repetitive motor response [76]. Given the length and duration of the test, sustained attention will be assessed [78, 79]. Children are instructed to cross out the target items, i.e., the dot patterns with four dots on a tablet covered in three, four, and five dot patterns. Dependent variables are performance and accuracy over time indicating fluctuations in attention. To assess search efficiency an object cancellation task was used. The presented screen contained 130 stimuli: red and green apples, green and brown pears, letters, and strings of letters. Children had to cancel all apples, while ignoring all other stimuli. Search efficiency will be measured including consistency of search direction, distance between two clicks, and number of intersections. The original capture task used in van der Stigchel & Nijboer was adapted to measure distractibility [80]. Each trial starts with the presentation of central fixation cross. When the cross disappeared, the target, represented as an apple, appeared in one of the corners. In 50 % of the trials, a distractor will appear as well. Reaction time will be measured for both conditions to calculate distractibility.

#### Psychosocial functioning

In children who are typical developing several studies proved a positive correlation between sports participation and self-perception [81]. To evaluate self-perception, the self-perception profile for children will be used [82]. This is a self-report scale that measures five specific domains of self-concept and the sense of general self-worth.

Children with physical disabilities who participate in sports had significantly higher health related quality of life satisfaction scores compared to their non-sporting peers [83]. The Disabkids questionnaire will be used to assess health related quality of life of children with chronic health conditions [84]. The questionnaire consists of 37 Likert-scaled items and is associated with three domains: mental, social and physical. Additionally, the three domains can be subdivided to six dimensions: independence, emotion, social inclusion, social exclusion, limitation, and treatment. The Disabkids has been developed and validated for children of age 4–16 years [85–87]. Older kids probably have not been included before, because some questions are related to school situations. Since all participants are still attending school, the questionnaire is considered to be applicable for all participants.

To establish the extent of participation in and adherence to a program of regular exercise or physical activity, the Exercise Self-Efficacy questionnaire scale (ESES) will be conducted [88]. A persons self-efficacy is largely influenced by past performances and accomplishments, or mastery experiences.

### Sample size

According to a study of Verschuren et al. an average decline of 10 ± 27 % in anaerobic fitness was calculated 3–4 months following a training program [23]. It has been hypothesized that participants following the sports program will show a difference of 20 % compared to the control group. The effect of 20 % seems realistic, since we previously found a 24 % increase in anaerobic fitness in children with cerebral palsy after a training program [23]. With an alpha of 0.05 and bèta of 0.20 (power of 0.80) a sample size of 32 subjects per group will be required. When taking a failure rate of 15 % into account, 74 subjects should be included in total in the Sport-2-Stay-Fit study.

### Statistical analyses

The effect of the HIT program and six months after-school sports program will be analysed using a multivariate repeated measures ANOVA. The possible differences between and within T0, T1 and T2 for the intervention group and control group will be calculated with a statistical significance level of *P* = 0.01. If there is a significant difference, a post-hoc test (a Bonferroni or LSD) will be executed to further investigate group differences. Quantitative descriptive statistics will be used to present demographics of the primary and secondary study parameters. All statistical analyses will be performed using SPSS for Windows (version 21.0, SPSS Inc, Chicago, Ill.) with a statistical significance level of *P* = 0.05.

## Discussion

The Sport-2-Stay-Fit study will provide insight in the effectiveness of an eight weeks HIT program in children and adolescents with a chronic disease or physical disability. Furthermore, the current study gives answer to the important question whether an after school sports program can increase the sustainability of the effectiveness of a HIT program. To our knowledge, this is the first study investigating the sustainability of a standardized training with a sport program in a group of children and adolescents with a chronic disease or physical disability.

In addition, sport participation in these children may have positive effects on cardiovascular health, injuries, and both cognitive and psychosocial functioning. While these positive relationships have been reported in healthy children, the benefits are not yet established in children with a chronic disease or physical disability.

The Sport-2-Stay-Fit study provide insight and understanding in both the effectiveness and feasibility of an after-school sport program following a standardized interval training. Changing physical activity during leisure time is complicated. Especially in children and adolescents with a chronic disease or physical disability who experience a lot of barriers in joining sport clubs. In this perspective, after-school sport participation may be a feasible solution to get those children and adolescents structurally active. The results are expected to be available in 2017.

## Abbreviations

HIT: high-intensity interval training
MPST: muscle power sprint test
SRT: shuttle run/ride test
VO_2_peak: peak oxygen uptake
VCO_2_: carbon dioxide production
RER: respiratory exchange ratio
BIA: bioelectrical impedance analysis
BMI: body mass index
PWV: pulse wave velocity
AIX: augmentation index
